# Nephrectomy as Salvage Treatment After an Unsuccessful Embolization of a Huge Incidental Renal Artery Aneurysm With an Arteriovenous Fistula

**DOI:** 10.7759/cureus.63565

**Published:** 2024-07-01

**Authors:** Panagiotis Katsikatsos, Periklis Anastasiou, Thomas Kalfas, Konstantinos Douroumis, Napoleon Moulavasilis, Sotirios Georgopoulos, Ioannis Anastasiou

**Affiliations:** 1 Department of Urology, National and Kapodistrian University of Athens, Athens, GRC; 2 Department of Urology, University of Ioannina, Ioannina, GRC; 3 Department of Vascular Surgery, Laikon General Hospital, Athens, GRC

**Keywords:** case report, nephrectomy, embolization, renal arteriovenous fistula, renal artery aneurysm

## Abstract

Renal artery aneurysms (RAA) with a concomitant renal arteriovenous fistula (RAVF) are rare entities with a reported incidence of less than 1%. An 86-year-old man was admitted to the urology department after an incidental finding of a left RAA on an abdominal ultrasound. A computed tomography angiography (CTA) revealed a saccular aneurysm measuring 54x42mm in the distal part of the left renal artery, along with a huge arteriovenous fistula measuring 45mm. The patient was asymptomatic at admission. Given the radiologic findings, an unsuccessful attempt at transcatheter arterial embolization was conducted. Therefore, a radical nephrectomy was recommended for the patient, which was performed without major intraoperative or postoperative complications. Microscopic examination depicted arteriosclerotic lesions and arterionephrosclerosis with 30% sclerotic glomeruli. The patient at his last visit remained free of symptoms. This case highlights the role of nephrectomy as a feasible option in cases of endovascular treatment failure.

## Introduction

Renal artery aneurysms (RAAs) are rare entities with a reported incidence of 0.01% to 1.3% [1,2]. They are usually asymptomatic and present as incidental findings in routine abdominal imaging like renal ultrasonography, computed tomography (CT), and magnetic resonance imaging (MRI). There has been a proposed categorization of renal aneurysms into three types by Rundback et al. according to the aneurysm morphology (saccular or fusiform) and the location in relevance to the main renal artery [3]. The treatment of RAAs depends on the type and consists of observation, endovascular techniques, and surgical repair. In the case of complex RAAs and the coexistence of arteriovenous fistulas, an open surgical treatment is proposed [2]. In this case report, we present RAA with an arteriovenous fistula, which was successfully treated with a nephrectomy after a failed transcatheter arterial embolization.

## Case presentation

An 86-year-old man was admitted to our urology department after a left RAA was discovered two months prior. The left RAA was found on an abdominal ultrasound scan performed as a screening test. The patient presented with no typical symptoms, like flank pain or hematuria. His medical history included hypertension, rheumatoid arthritis, and glaucoma, for which he was on medication. His surgical history was notable for a laparoscopic cholecystectomy three years ago, transurethral resection of the prostate for benign prostatic hyperplasia (BPH), and hernia repair surgery. His family history was remarkable for hypertension on both the paternal and maternal sides.

**Figure 1:**
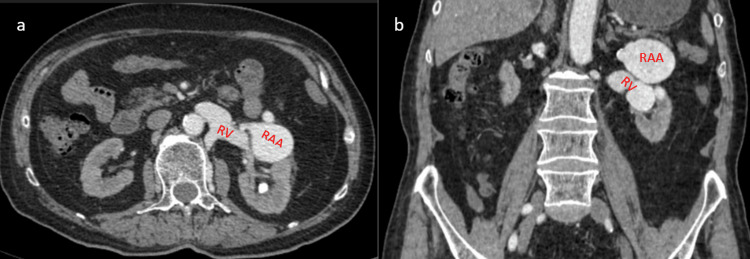
a) The axial plane of computed tomography angiography (CTA) shows a left renal artery aneurysm (RAA) measuring 54x42mm in relation to the left renal vein (RV). b) The coronal plane of the CTA depicts the dilation of the RV in the arterial phase, indicating the arteriovenous fistula (RAVF).

A CT angiography (CTA), following the ultrasound finding, depicted a saccular aneurysm measuring 54x42mm in the distal part of the left renal artery concomitant with a huge renal arteriovenous fistula (RAVF) measuring 45mm in maximal diameter, below the aneurysm at the level of the middle and lower pole of the left kidney in relation to the left renal vein (Figure 1). There was also a right renal artery aneurysm as well as a splenic artery aneurysm.

**Figure 2:**
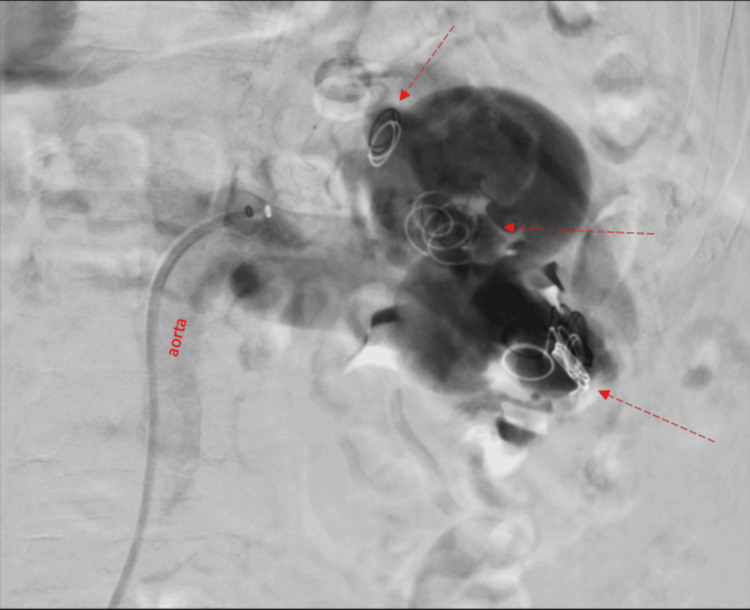
Angiography shows the renal artery aneurysm and several coils (arrows) in the aneurysm and in the renal vein after the unsuccessful embolization.

A review of the diagnostic findings was presented at a multidisciplinary board meeting composed of a urologist, a vascular surgeon, and an interventional radiologist. Treatment options included endovascular techniques, surgical repair of the aneurysm and RAVF, and nephrectomy. Firstly, an angiography was conducted, and a transcatheter arterial embolization was performed unsuccessfully (Figure 2). Therefore, surgical treatment by nephrectomy was recommended for the patient.

**Figure 3:**
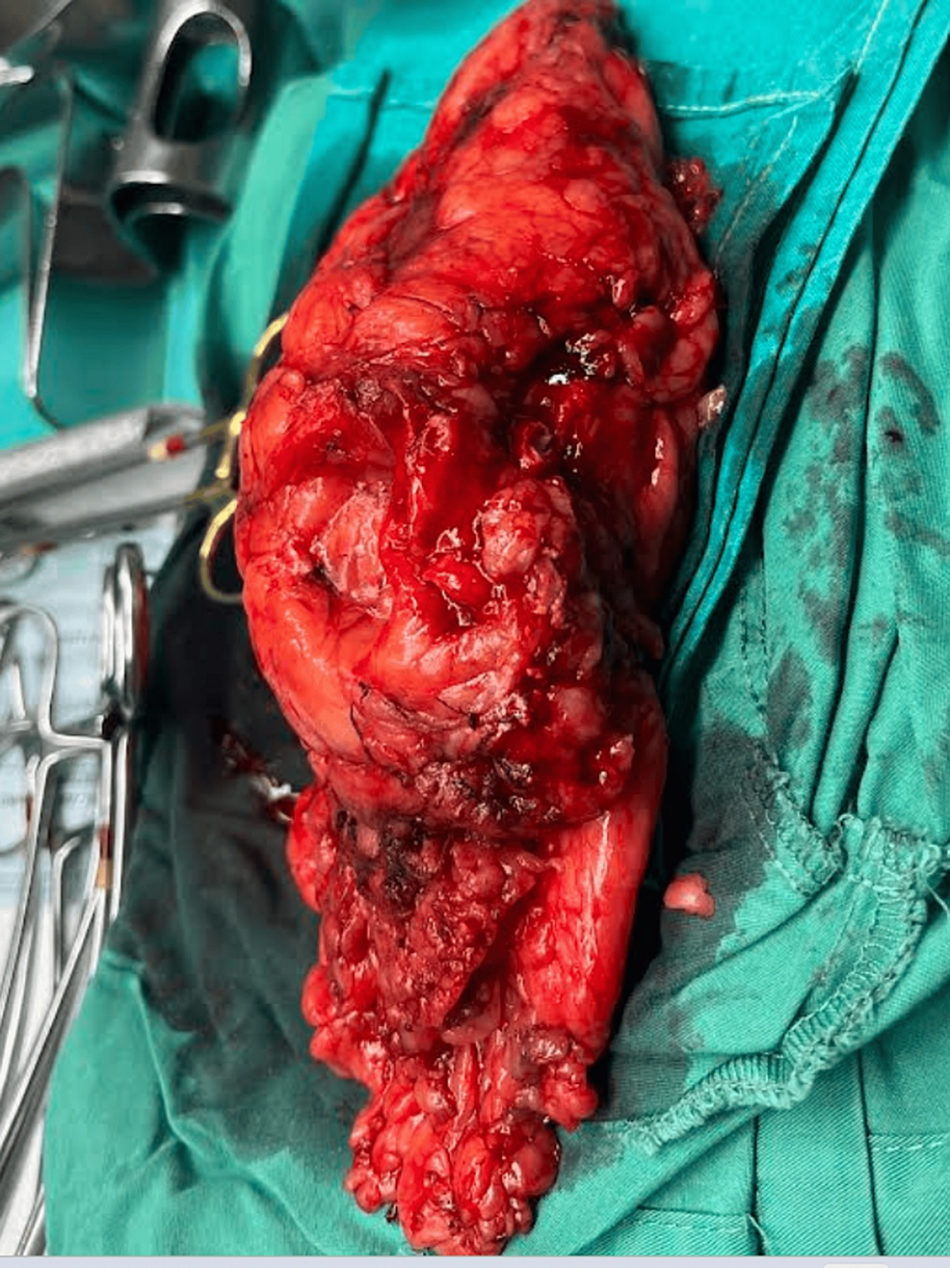
Intraoperative photo of the excised left kidney

A left radical nephrectomy was performed under general anesthesia. The patient was placed in a modified flank position. The nephrectomy was performed without major intraoperative complications, and the kidney was excised along with Gerota’s fascia (Figure 3). The operation time was 106 minutes. The total blood loss was 350 cc. The specimen was sent for pathologic analysis.

The postoperative course was uneventful. The patient was closely monitored during the first two postoperative days. On postoperative day three, the drainage tube was removed, and on postoperative day five, the Foley catheter was removed, and the patient was discharged. Pathologic evaluation of the specimen revealed a 4 cm RAA with a direct renal artery-to-renal vein connection. Microscopically, the specimen showed arteriosclerotic lesions and arterionephrosclerosis with 30% sclerotic glomeruli. During his last visit, three months after the operation, the patient was well and remained free of symptoms. His creatinine level was 1.6 mg/dL with an estimated glomerular filtration rate (eGFR) of 42 ml/min/1.73 m².

## Discussion

A renal artery aneurysm is defined as a dilation that is twice the size of a regular renal artery’s diameter [4]. The occurrence of RAA in patients who have had renal angiography performed varies from 0.3% to 1% in published studies. They are usually asymptomatic; however, symptoms associated with RAA are hematuria, flank pain, palpable abdominal mass, and hypertension [5]. Rundback et al. have proposed a classification system for RAA according to the aneurysm morphology and location in relevance to the main renal artery, consisting of three types. Type 1 aneurysms are saccular, arising from the main renal artery or the proximal branches of the artery. Type 2 aneurysms occur at the main renal artery or proximal segmental arteries and are fusiform-shaped. Type 3 aneurysms are detected intraparenchymally and occur in the small segmental arteries [3]. In this case, the patient had a type 1 RAA measuring 5.4 cm in maximal diameter.

Management options for RAA include a conservative approach or surgical treatment. Conservative treatment is indicated in asymptomatic patients and aneurysms with a diameter of less than 2 cm. Surgical treatment options vary, with nephrectomy, vascular bypass, nephrectomy with ex vivo angioplasty, autotransplantation, endovascular treatment like coil embolization, and stent-graft placement being preferred if technically feasible. Indications for surgical treatment include pregnant women or those who wish to be pregnant, rapid size expansion with a diameter greater than 2 cm, RAA associated with RAVF, symptomatic aneurysms, and rupture. The surgical method to be chosen depends on the type of aneurysm according to Rundback’s classification [6].

Renal arteriovenous fistulas are another rare entity and may be associated with RAAs. Renal arteriovenous anomalies consist of two types: the aneurysmal type, in which a single communication between the artery and the vein is found, and the cirsoid type, which consists of multiple arteriovenous communications [4]. The aneurysmal type is further subdivided into idiopathic and secondary. Idiopathic RAVFS are considered to be created due to the pressure of the aneurysmal main renal artery or segmental artery on the adjacent vein. Secondary RAVFS are created after interventions in the area of the kidney, such as trauma or percutaneous surgical procedures. Cirsoid types are considered congenital malformations between the artery and the vein [7]. Idiopathic RAVFS are considered exceedingly rare, with an incidence of 2.8% of all fistulas. The clinical presentation varies from completely asymptomatic to abdominal bruit, congestive heart failure, hypertension, and hematuria [8].

Both RAAs and RAVFS can be diagnosed and evaluated by duplex ultrasound, spiral CT, and digital subtraction arteriography (DSA). Digital subtraction arteriography is deemed the gold standard both for highlighting details and for choosing the appropriate method of intervention [4]. In small or asymptomatic fistulas, conservative treatment is indicated. Operative management options include nephrectomy, ex vivo bench repair, and endovascular techniques. Ex vivo bench repair and vascular reconstruction are surgical options that offer the advantage of functional kidney preservation. Transcatheter embolization has become the gold standard for RAVFS in recent years due to the less invasive and kidney-sparing nature of this technique [6, 9-11].

In our case, a renal artery aneurysm of type 1, measuring above 5 cm in its maximal diameter, was combined with a huge arteriovenous fistula measuring 4.5 cm. The patient reported no history of trauma or intervention in the genitourinary system, so the RAVF was deemed idiopathic. After an angiography was performed, which confirmed the findings of the CT scan, we strived for arterial embolization in an attempt to be less invasive and preserve the maximum of the patient's renal function. This effort was unsuccessful, so nephrectomy was determined to be the optimal alternative. The uneventful recovery, the good condition of the patient at the three-month follow-up, and the stable creatinine levels demonstrate that nephrectomy remains a feasible option in cases of endovascular treatment failure or ineligibility.

## Conclusions

Renal artery aneurysms and RAVFs are rare but significant vascular anomalies that require careful diagnostic and therapeutic approaches. This case highlights the complexity and interrelationship of RAAs and RAVFs. The successful treatment through nephrectomy underscores the importance of a multidisciplinary approach in managing these conditions, especially when conservative and less invasive methods, such as transcatheter arterial embolization, fail. The postoperative outcome of the patient demonstrates that, despite the invasiveness of nephrectomy, it can be an effective treatment option with a satisfactory recovery and prognosis.
